# Sitting Baduanjin-based rehabilitation programme following endovascular therapy in patients with chronic limb-threatening ischemia: protocol for a WeChat mini program-assisted multicentre randomized controlled trial

**DOI:** 10.3389/fcvm.2026.1921616

**Published:** 2026-10-09

**Authors:** Jiayi Song, Li Fu, Xiaomei Fang, Haiyan Chen, Lian Wang, Liang Xing, Huanping Liu, Yun Xu

**Affiliations:** 1 Department of Oncology, Xiyuan Hospital of China Academy of Chinese Medical Sciences, Beijing, China; 2 Department of Peripheral Vascular Surgery, Xiyuan Hospitall of China Academy of Chinese Medical Sciences, Beijing, China; 3 Department of Peripheral Vascular Surgery, Guang'anmen Hospital China Academy of Chinese Medical Sciences, Beijing, China; 4 Department of Peripheral Vascular Surgery, Wang Jing Hospital China Academy of Chinese Medical Sciences, Beijing, China

**Keywords:** chronic limb-threatening ischemia, endovascular therapy, Sitting Baduanjin, study protocol, WeChat mini program

## Abstract

**Background:**

Endovascular therapy (EVT) is an important revascularization strategy for limb salvage in patients with chronic limb-threatening ischemia (CLTI). However, despite restoration of blood flow, patients may continue to experience residual haemodynamic impairment and ischaemia-related burden after EVT, with a persistently high risk of adverse limb outcomes. Therefore, effective post-EVT rehabilitation strategies are needed to further improve limb perfusion and promote functional recovery and symptom improvement in this population. Exercise rehabilitation is widely recommended for patients with peripheral artery disease (PAD), but current evidence is largely derived from individuals with intermittent claudication, and its applicability and additional benefits in patients with CLTI after EVT remain uncertain. Sitting Baduanjin (SBDJ) is a low-intensity mind–body exercise modality that reduces lower-limb loading and postural demands while providing exercise stimulation, with potential cardiovascular regulatory effects, and may therefore be suitable for patients with limited exercise tolerance after EVT. However, evidence regarding the effects of SBDJ on target-limb haemodynamic status and patient-centred outcomes in patients with CLTI following EVT remains limited.

**Methods:**

This prospective, multicentre, parallel-group randomised controlled trial incorporates blinded outcome assessment. A total of 146 eligible patients with CLTI following EVT will be randomly assigned in a 1:1 ratio to the intervention group or the control group. The intervention group will receive SBDJ combined with ankle pump exercise, whereas the control group will receive ankle pump exercise. A WeChat mini program will support exercise delivery, adherence monitoring, follow-up management, and questionnaire data collection during the home-based phase. The primary outcome is the longitudinal change in target-limb ABI over the 12-week rehabilitation period. Secondary outcomes include walking ability, quality of life, anxiety symptoms, lower-limb rest pain, and safety. Outcomes will be assessed at baseline, week 4, and week 12.

**Discussion:**

This trial aims to determine whether an SBDJ-based rehabilitation programme provides additional benefits over ankle pump exercise alone on index-limb haemodynamic status among patients with CLTI following EVT, while exploring its potential effects on functional outcomes and patient-reported outcomes during the early post-EVT rehabilitation period.

**Clinical Trial Registration:** http://itmctr.ccebtcm.org.cn/, identifier: ITMCTR ITMCTR2022000113.

## 1 Background

Chronic limb-threatening ischemia (CLTI) represents the most advanced manifestation of lower-extremity peripheral artery disease (PAD) and is clinically characterized by ischemic rest pain, nonhealing ulcers, or gangrene (1). Without timely intervention, CLTI may progress to major amputation, sepsis, and mortality. Endovascular therapy (EVT) has become the primary revascularization strategy for limb salvage by restoring perfusion to the ischemic limb (2). However, EVT successfully restores arterial flow, but does not fully reverse the functional and haemodynamic consequences of CLTI. Patients following EVT remain at risk of recurrent ischaemia, wound-related complications, and adverse limb events, with a reported 4-year survival rate of only 38% (3). In addition, CLTI imposes a substantial healthcare burden, with the annual treatment cost per patient reported to be approximately $49,700 (4). Therefore, optimising post-EVT management strategies to improve lower limb perfusion and long-term clinical outcomes remains an important unmet need in the management of vascular disease.

Exercise rehabilitation has increasingly gained attention in the management of atherosclerotic vascular diseases. Supervised exercise therapy (SET) is recommended by clinical guidelines as an important component of conservative management for patients with PAD. However, the additional benefits of exercise therapy beyond standard care following lower limb revascularisation remain uncertain, including regarding vascular-related outcomes and patient-centred outcomes (5). Moreover, evidence supporting exercise rehabilitation in patients with CLTI during the early period after EVT remains limited. Although revascularisation restores patency of occluded peripheral arteries, ischaemia-related abnormalities, including microcirculatory dysfunction, vascular impairment, and skeletal muscle impairment, may persist and contribute to residual functional limitations and impaired limb haemodynamic status.

Patients with CLTI following EVT may continue to experience wound-related limitations, reduced mobility, and a substantial burden of cardiovascular and metabolic comorbidities, which may restrict participation in conventional walking-based exercise programmes (6). Therefore, there is a need to explore safe, feasible, and low-burden exercise modalities tailored to this vulnerable population. Baduanjin is a traditional Chinese Qigong exercise. Unlike conventional aerobic or resistance exercises that primarily aim to enhance physical capacity, Baduanjin is a mind-body exercise characterized by slow and fluid movements, mental focus, and controlled breathing. Sitting Baduanjin (SBDJ) is an adapted form of Baduanjin that modifies the traditional standing practice into a seated format, thereby reducing lower-limb loading and postural balance requirements while preserving the characteristic movement sequences and breathing components (7, 8). These characteristics may make SBDJ particularly suitable for patients with CLTI following lower limb EVT. Previous studies have demonstrated the feasibility and safety of SBDJ and suggested potential cardiovascular benefits, including favourable effects on arterial stiffness, arterial elasticity, and blood pressure regulation. In addition, the reported effects of SBDJ on cardiopulmonary function and autonomic regulation suggest broader cardiovascular regulatory effects. However, it remains unclear whether these systemic cardiovascular effects can contribute to improvements in target-limb haemodynamic status and clinical outcomes in patients with CLTI following EVT.

Furthermore, the implementation of exercise rehabilitation in routine clinical practice remains challenging due to barriers such as transportation difficulties, time constraints, financial burden, and insufficient ongoing supervision. Advances in digital health technologies provide new opportunities to extend rehabilitation beyond clinical settings (9). Accordingly, we developed a WeChat Mini Program-based digital support platform to facilitate SBDJ implementation, adherence management, and remote follow-up after discharge. This study aimed to investigate whether a digitally supported SBDJ rehabilitation programme could improve target-limb haemodynamic status in patients with CLTI following lower-limb EVT and to explore its potential effects on walking ability, quality of life, lower-limb rest pain, and anxiety symptoms.

## 2 Methods

### 2.1 Study design

This prospective, multicentre, parallel-group randomised controlled trial with blinded outcome assessment was developed according to the Standard Protocol Items: Recommendations for Interventional Trials (SPIRIT) guidelines and will be conducted in accordance with the Declaration of Helsinki. A total of 146 eligible patients will be randomly assigned to either the intervention group, receiving SBDJ in addition to ankle pump exercise, or the control group, receiving ankle pump exercise alone. Both groups will receive a 12-week intervention, with outcome assessments performed at baseline, week 4, and week 12. The schedule of enrolment, interventions, and assessments is presented in Table 1.

**Table 1:** SPIRIT schedule of enrollment, interventions, and assessments.

Study period	Enrollment	Allocation	Post-allocation												Close-out
Time point	−t1	T0				T1								T2	After T2
	During the postoperative hospital stay	Week 0	Week 1	Week 2	Week 3	Week 4	Week 5	Week 6	Week 7	Week 8	Week 9	Week 10	Week 11	Week 12	
Enrollment															
Identification of potential participants	X														
Eligibility screening	X														
Written informed consent	X														
Allocation															
Baseline assessment		X													
Center-stratified randomization with concealed allocation		X													
Interventions															
Intervention group: Sitting Baduanjin combined with ankle pump exercise			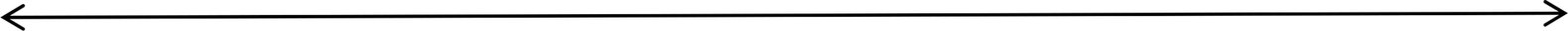												
Control group: ankle pump exercise			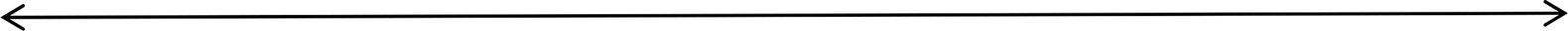												
Assessments															
Demographic and clinical characteristics		X													
ABI		X				X								X	
WHOQOL-BREF		X				X								X	
WIQ		X				X								X	
SAS		X				X								X	
NRS		X				X								X	
Adverse event monitoring			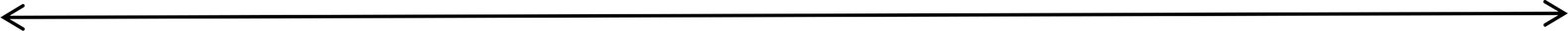												
Close-out															
Data collection and statistical analysis															X

X indicates that the item is performed at the specified time point; → indicates that the item is continued throughout the specified period. Randomization will be performed after completion of the baseline assessment at T0. T0 refers to the baseline assessment; T1 refers to the week 4 assessment during the intervention period; and T2 refers to the week 12 assessment at the end of the intervention period. ABI, ankle-brachial index; WHOQOL-BREF, World Health Organization Quality of Life-BREF; WIQ, Walking Impairment Questionnaire; SAS, Self-Rating Anxiety Scale; NRS, Numerical Rating Scale. Both groups will receive usual postoperative care during hospitalization and standardized discharge education before discharge.

The study protocol has been approved by the Ethics Committee of Xiyuan Hospital, China Academy of Chinese Medical Sciences (approval number: 2022XLA078-2; approved on 2 June 2022) and has been prospectively registered with the International Traditional Medicine Clinical Trial Registry (ITMCTR; identifier: ITMCTR2022000113; registered on 29 June 2022). The overall study flow diagram is presented in Figure 1.

**Figure 1:**
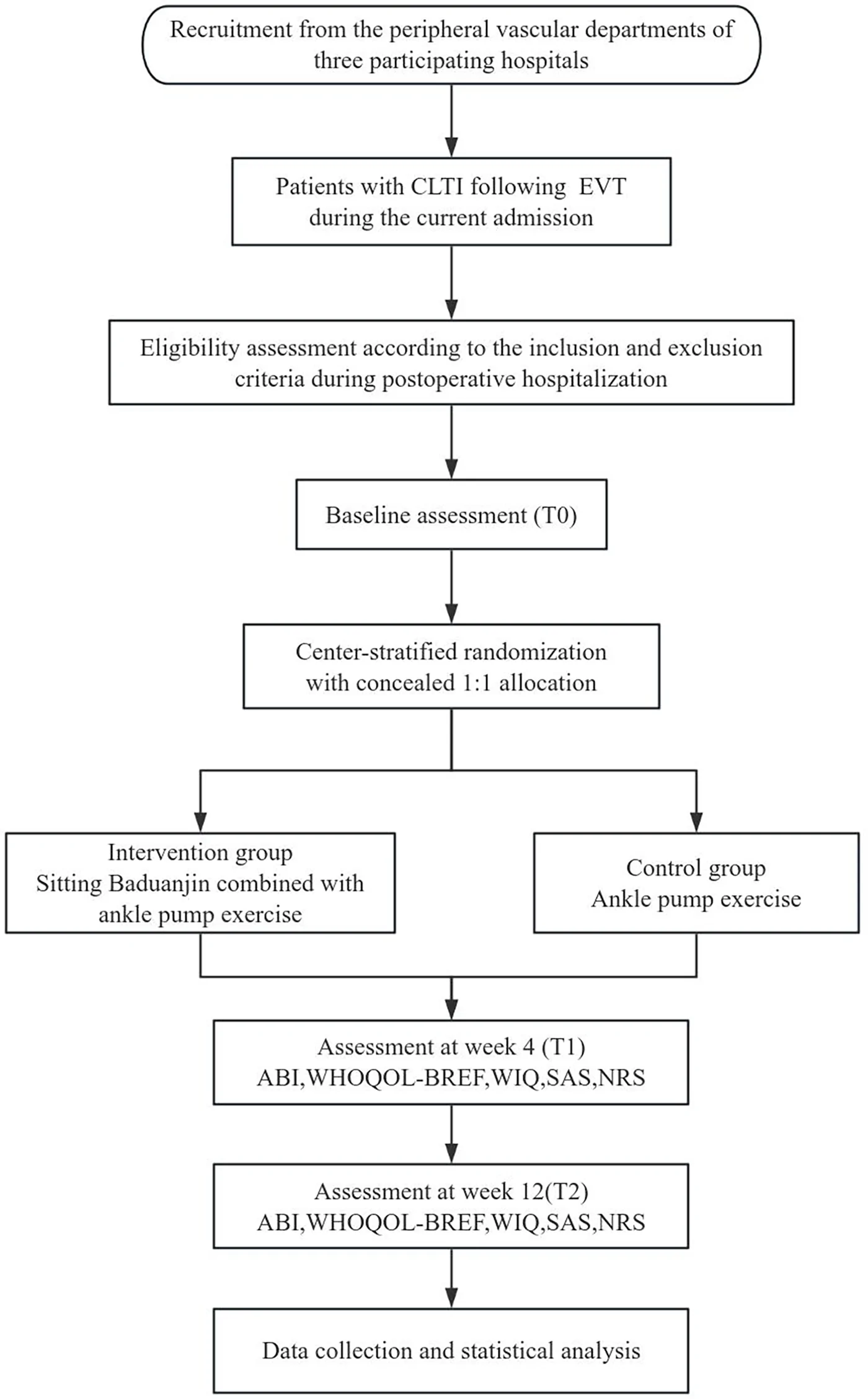
The flow chart of the trial design. ABI, ankle-brachial index; WHOQOL-BREF, World Health Organization Quality of Life-BREF; WIQ, Walking Impairment Questionnaire; SAS, Self-Rating Anxiety Scale; NRS, Numerical Rating Scale.

### 2.2 Participants and recruitment

Participant recruitment will commence in December 2022 across the Departments of Peripheral Vascular Disease at Xiyuan Hospital, Guang'anmen Hospital, and Wangjing Hospital, China Academy of Chinese Medical Sciences. Potentially eligible inpatients with CLTI will be identified through review of procedural records by ward nurses and referred to research assistants for preliminary assessment. Subsequently, two trained research assistants will independently perform eligibility assessments according to predefined inclusion and exclusion criteria during the postoperative hospital stay following lower-limb EVT.

#### 2.2.1 Inclusion criteria

Patients will be eligible if they meet all of the following criteria:

1. Diagnosis of CLTI according to the *Global Vascular Guidelines on the Management of Chronic Limb-Threatening Ischemia*;
2. Underwent lower-limb EVT during the current hospitalisation;
3. Age 18–75 years, regardless of sex;
4. Provided written informed consent and voluntarily agreed to participate in the trial.

#### 2.2.2 Exclusion criteria

Patients will be excluded if they meet any of the following criteria:

1. Target-limb manifestations primarily attributable to non-atherosclerotic or non-ischaemic conditions, including venous ulceration, traumatic injury, acute limb ischaemia, arterial embolism, vasculitis, thromboangiitis obliterans (Buerger disease), or radiation arteritis;
2. Ischaemic wounds involving more than 20% of the total foot area;
3. Unsuccessful index endovascular revascularisation of the target limb, as documented in the endovascular procedure report;
4. Severe, uncontrolled, or clinically unstable cardiovascular, cerebrovascular, hepatic, renal, haematological, or endocrine diseases;
5. Severe hearing or visual impairment, communication difficulties, moderate-to-severe cognitive impairment, or uncontrolled psychiatric disorders.

#### 2.2.3 Criteria for discontinuation of study intervention

The assigned intervention will be discontinued under the following circumstances:

1. Repeat ipsilateral target-limb revascularisation or other limb-related procedures that prevent continuation of the assigned exercise intervention;
2. Serious adverse events or clinically significant medical conditions, including cardiovascular or cerebrovascular events, that render continuation of the intervention unsafe or inappropriate;
3. Major or minor amputation or other medical conditions that prevent continuation of the assigned exercise intervention;
4. Participant request to discontinue the assigned intervention.

Discontinuation of the intervention will not be considered withdrawal from the trial. Unless informed consent is withdrawn, participants will continue follow-up assessments whenever possible.

### 2.3 Informed consent

Eligible patients will be approached by trained research assistants after clinical stabilisation and confirmation of their ability to provide informed consent. Participants will receive oral and written information regarding the study objectives, procedures, randomisation, interventions, potential risks and benefits, and their right to withdraw without affecting subsequent medical care. They will have sufficient time to consider participation and discuss any questions with the research team. Written informed consent will be obtained before enrolment, randomisation, and initiation of any study-related procedures.

### 2.4 Randomization and allocation

Participants will be randomly assigned in a 1:1 ratio to the intervention group or control group using a centralised allocation system. An independent statistician who is not involved in participant recruitment, intervention delivery, outcome assessment, or data analysis will generate the allocation sequence using SAS software. Randomisation will be performed using a centre-stratified permuted block design with variable block sizes.

Allocation concealment will be maintained by an independent randomisation administrator at the trial coordinating centre, who will retain the allocation sequence and block sizes. After eligibility confirmation, written informed consent, and completion of baseline assessment, site research assistants will contact the central randomisation administrator for group assignment. The administrator will release the assignment according to the concealed sequence corresponding to the participant's study centre. Randomisation details, including participant ID, study centre, date and time of randomisation, and assigned group, will be recorded in the randomisation log.

### 2.5 Blinding

Due to the nature of the exercise intervention, participants and intervention providers cannot be blinded to group allocation. Outcome assessors responsible for ABI measurement will be blinded to group allocation to minimise detection bias. Patient-reported outcomes will be assessed using validated questionnaires according to predefined procedures. Data managers and trial statisticians will remain blinded throughout data management and statistical analysis to minimise potential analytical bias. Any accidental unblinding will be documented and evaluated for its potential impact on study integrity.

### 2.6 Implementation

All participants will receive standardized postoperative care, including routine medical management, health education, and discharge instructions. Exercise interventions will be initiated during hospitalization after postoperative stabilization. Before exercise initiation, patients will be assessed to confirm stable vital signs and the absence of active bleeding, hematoma, or oozing at the puncture site. The research team will provide in-hospital exercise instruction. After discharge, participants will continue protocol-based home-based training with assistance from the WeChat mini program.

#### 2.6.1 Control group

Participants randomized to the control group will receive ankle pump exercise training alone during the 12-week intervention period. During hospitalization, rehabilitation physicians will provide instruction on ankle pump exercises and correct improper movements when necessary. After discharge, participants will continue ankle pump exercise at home with access to standardized exercise videos through the WeChat mini program.

The prescribed ankle pump exercise regimen consists of three sessions per day, with each session lasting approximately 3–5 min. During the intervention period, participants in the control group will not receive SBDJ-related training videos or instructional materials to avoid additional exercise exposure that may influence study outcomes. After completion of the final follow-up assessment, participants may access SBDJ-related materials upon request.

#### 2.6.2 Intervention group

Participants randomized to the intervention group will receive a 12-week SBDJ-based rehabilitation programme, which incorporates SBDJ training in addition to the same ankle pump exercise regimen received by the control group.

A standardized SBDJ protocol was developed based on established Baduanjin training procedures under the guidance of experts in traditional Chinese medicine rehabilitation and exercise therapy. The protocol was adapted to the functional status and exercise tolerance of patients with CLTI following EVT to improve safety, feasibility, and intervention fidelity across participating centers. Based on previous studies evaluating exercise intensity, SBDJ is considered a light-intensity mind–body exercise modality (10–12). The Borg Rating of Perceived Exertion (RPE) scale will be used to standardize and monitor exercise intensity during training sessions, with the target intensity maintained at approximately 9–11 on the 6–20 Borg scale.

During hospitalization, participants will receive face-to-face small-group SBDJ training (≤8 participants per group), delivered by three designated nurses at each participating center who received standardized training. These nurses will be responsible for SBDJ instruction, training delivery, and quality supervision. The training includes standardized instructional video demonstrations, movement sequence instruction, safety precautions, and correction of incorrect movements.

The SBDJ programme consists of eight standardized movements, with each movement repeated 4–8 times. Each training session lasts approximately 15 min. Participants will be encouraged to practice SBDJ once daily, with a minimum frequency of three sessions per week.

After discharge, participants will continue home-based SBDJ combined with ankle pump exercise training, supported by the WeChat mini program, which provides standardized exercise guidance.

#### 2.6.3 WeChat mini program-assisted intervention delivery and adherence monitoring

To support home-based rehabilitation after discharge, the research team developed the “CLTI Exercise Rehabilitation Guidance” WeChat mini program based on the study protocol. The platform supports standardized exercise guidance, training documentation, health education, and remote follow-up for participants in both groups.

The WeChat mini program serves as a digital rehabilitation management platform to upport delivery of the prescribed exercise protocols and monitoring of exercise adherence. All participants will use the platform to access exercise guidance, record training completion, and complete study-related assessments. The platform includes four modules: exercise check-ins, training reminders, health education, and study questionnaires (Figure 2).

**Figure 2:**
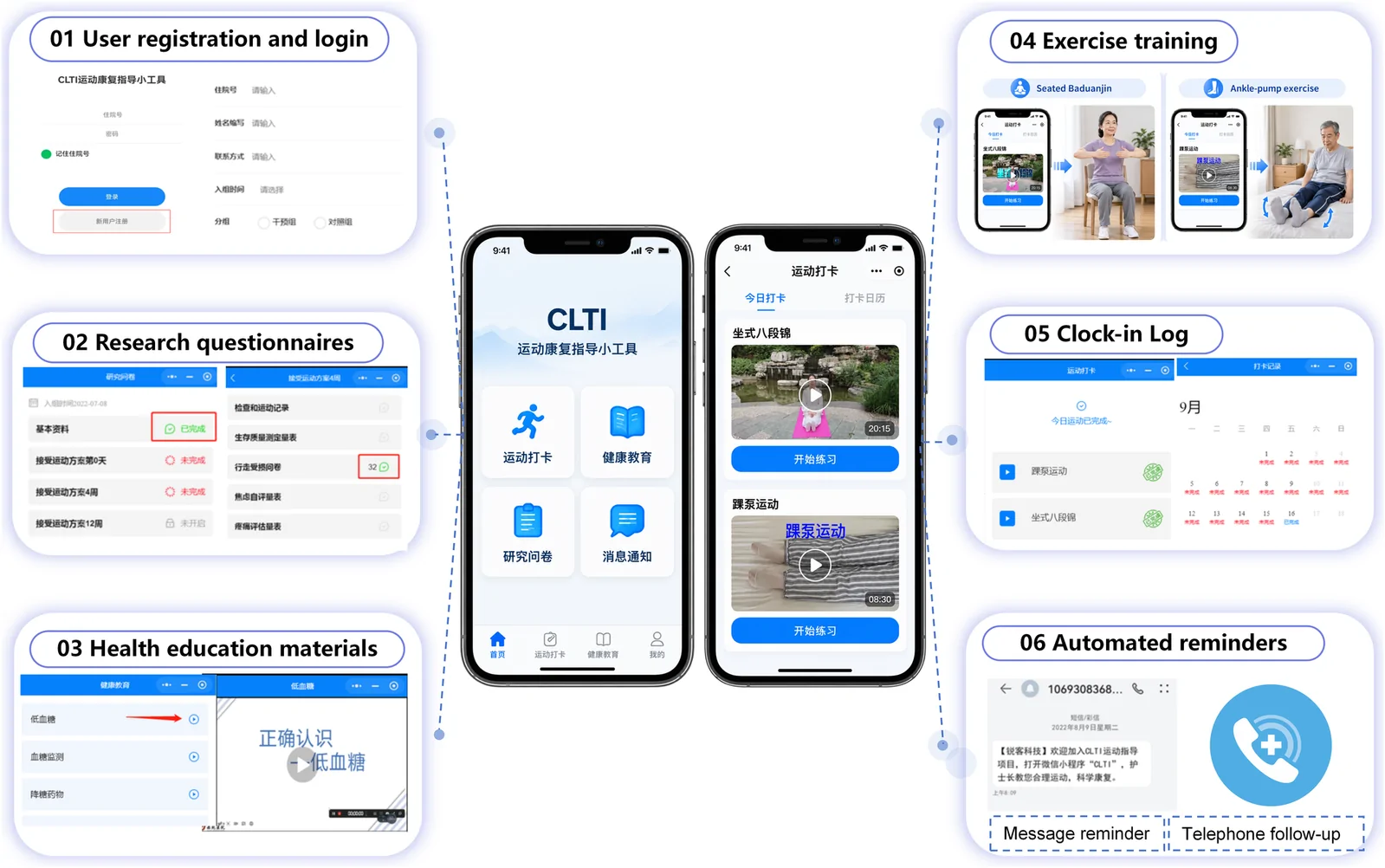
Functional modules of the CLTI exercise rehabilitation guidance WeChat mini program.

The exercise check-in module provides group-specific exercise videos according to randomization allocation and generates electronic training logs after completion of the guided sessions. The training reminder module delivers scheduled reminders through WeChat notifications and SMS messages. The health education module provides structured educational materials related to CLTI management and postoperative self-care. The study questionnaire module enables participants to complete scheduled patient-reported questionnaires during follow-up.

Before discharge, adherence managers will assist participants with registration and provide instructions on platform use. During home-based rehabilitation, exercise records will be reviewed regularly. Participants with no recorded exercise completion for three consecutive days will receive telephone follow-up to identify barriers and provide appropriate support. Exercise completion may be reported by telephone and documented by the research team for participants unable to complete electronic records due to technical difficulties.

### 2.7 Exercise adherence assessment

Exercise adherence will be assessed throughout the 12-week intervention period based on completion of prescribed exercise sessions. Adherence will be calculated as the proportion of completed sessions relative to the scheduled sessions. During hospitalization, attendance at exercise instruction or training sessions will be documented using standardized records. After discharge, completion of home-based exercise sessions will be assessed based on electronic training records generated through the WeChat mini program. For participants unable to complete electronic records due to technical difficulties, exercise completion will be collected through telephone follow-up and documented by the research team.

Participants completing ≥80% of each prescribed exercise component will be considered to have adequate adherence. The adherence criteria are defined as follows: ≥68 of 84 planned SBDJ sessions and ≥202 of 252 planned ankle pump exercise sessions in the intervention group, and ≥202 of 252 planned ankle pump exercise sessions in the control group. Reasons for missed sessions will be documented and summarized.

### 2.8 Harms and adverse events

Participants experiencing discomfort during exercise will be instructed to stop training and contact the research team. All adverse events (AEs) occurring during the study period will be recorded regardless of their relationship to the intervention. The onset, severity, potential relationship to the intervention, management, and outcome of each AE will be documented in the CRF. Serious adverse events (SAEs), including death, life-threatening events, hospitalization or prolonged hospitalization, significant disability, or other medically important events, will be recorded separately and reported to the ethics committee within 24 h of identification.

### 2.9 Outcome

Both groups will undergo standardised outcome assessments at baseline (T0), week 4 (T1), and week 12 (T2). Outcomes will include target-limb ABI, quality of life, walking ability, anxiety symptoms, and lower-limb rest pain.

#### 2.9.1 Primary outcome

The ankle-brachial index (ABI) is a widely used, non-invasive measure of lower-extremity arterial haemodynamics in patients with peripheral artery disease (PAD). ABI reflects arterial haemodynamic impairment and provides an indirect assessment of limb perfusion status (13). Following lower-extremity revascularisation, current guidelines recommend ABI assessment to monitor changes in limb haemodynamics and perfusion after intervention (14). Therefore, ABI was selected as the primary outcome to assess changes in target-limb haemodynamic status during the 12-week post-EVT rehabilitation period.

The limb undergoing lower-limb EVT during the index hospitalisation was defined as the index limb. For participants undergoing bilateral EVT, the limb with the more severe Rutherford category was selected as the index limb. If both limbs showed comparable severity, the limb with the lower baseline ABI was designated as the index limb. ABI measurements will be performed at the research centre by trained assessors using an automated peripheral vascular assessment system (ANGIOLAB2, ATYS Medical, France) according to a standardised protocol. Participants will rest in the supine position for 10 min before measurement. The device will automatically measure bilateral brachial systolic pressures and ankle systolic pressures at the dorsalis pedis and posterior tibial arteries of the index limb.

Medial arterial calcification, which is commonly observed among patients with diabetes, older age, or chronic kidney disease, may impair arterial compressibility and affect ABI interpretation. ABI values >1.40, indicating non-compressible arteries, will therefore be recorded separately and considered unreliable for haemodynamic assessment (14).

#### 2.9.2 Secondary outcomes

##### 2.9.2.1 World health organization quality of life-BREF

Quality of life will be assessed using the Chinese version of the World Health Organization Quality of Life-BREF (WHOQOL-BREF). The WHOQOL-BREF is a 26-item generic patient-reported outcome measure covering four domains: physical health, psychological health, social relationships, and environment (15). Each item is rated on a 5-point Likert scale ranging from 1 (e.g., very dissatisfied) to 5 (e.g., very satisfied). Domain scores are calculated according to the established scoring algorithm, with transformed scores ranging from 0 to 100. Higher scores indicate better self-perceived quality of life (16). No established MCID is available for WHOQOL-BREF in patients with CLTI following EVT.

##### 2.9.2.2 Walking impairment questionnaire

Walking ability will be assessed using the Chinese version of the Walking Impairment Questionnaire (WIQ) (17). The WIQ is a PAD-specific patient-reported outcome measure that assesses disease-related walking limitations in daily activities, including self-perceived limitations in walking distance, walking speed, and stair-climbing ability. It provides complementary information regarding real-world mobility restriction during post-EVT rehabilitation (18–20). The questionnaire consists of 21 items rated on a 5-point Likert scale, with response options ranging from no difficulty to inability to perform the activity (21). Scores will be calculated according to the scoring instructions and transformed to a 0–100 scale, with higher scores indicating better self-reported walking ability. Although an established MCID has not been determined for WIQ scores, a previous study in patients with PAD undergoing exercise rehabilitation suggested that an improvement of approximately 5 points in the WIQ distance score may represent a small clinically meaningful change (22).

##### 2.9.2.3 Self-rating anxiety scale

Anxiety symptoms will be assessed using the Zung Self-Rating Anxiety Scale (SAS). The SAS consists of 20 items assessing anxiety-related symptoms across four domains: cognitive, autonomic, motor, and central nervous system symptoms. Each item is rated on a 4-point Likert scale ranging from 1 (“not at all”) to 4 (“most or all of the time”), resulting in a total raw score ranging from 20 to 80. Higher scores indicate greater anxiety severity (23). No established MCID is available for SAS scores in patients with CLTI following EVT.

##### 2.9.2.4 Numeric rating scale

Lower-limb rest pain will be assessed using the Numeric Rating Scale (NRS) (24). Participants will rate the intensity of ischemic rest pain in the revascularized limb using an 11-point scale ranging from 0 (“no pain”) to 10 (“worst imaginable pain”). Higher scores indicate greater pain severity. A reduction of ≥2 points or ≥30% from baseline will be considered a clinically meaningful improvement in pain intensity based on established criteria for interpreting changes in pain severity.

### 2.10 Data collection and management

Study data will be collected and managed using case report forms (CRFs) and an electronic data platform.

At baseline, demographic characteristics (age, sex, education level, and smoking status), clinical characteristics (diabetes, hypertension, coronary heart disease, cerebrovascular disease, hyperlipidemia, and other comorbidities), disease severity indicators (Rutherford classification, wound status, and wound size), and EVT-related procedural information (treated vascular segments, treated vessels, extent of vascular involvement, and treatment approach) will be collected. Data will be obtained from medical records, procedural records, and participant interviews as appropriate, and documented in CRFs by study evaluators.

During follow-up, patient-reported outcome data will be collected primarily through the WeChat mini program. For participants unable to use the platform, evaluators will conduct scheduled telephone interviews using the same standardized assessment tools and record responses in CRFs. Data managers will be responsible for data entry, export, verification, and quality control. Data from CRFs and the WeChat mini program backend will be integrated into a centralized electronic database using unique participant identification numbers. After data cleaning and consistency checks, the database will be locked before statistical analysis.

### 2.11 Sample size

We performed an *a priori* power analysis using the web-based GLIMMPSE software based on a general linear mixed-model framework (25). The significance level was set at 0.05, with 80% power. The design included two groups (SBDJ and control groups), three repeated measurements (T0, T1, and T2), and a 1:1 allocation ratio. No previous study has directly evaluated the effect of SBDJ in patients with CLTI following EVT. Therefore, the anticipated treatment effect was informed by previous exercise-based studies in patients with PAD. A randomized controlled trial in patients with PAD reported an approximately 0.08 between-group difference in ABI change after exercise training (26). In the absence of direct evidence regarding the timing of ABI changes following exercise intervention, we assumed a gradual accumulation of the treatment effect over the 12-week follow-up period, with half of the anticipated effect specified at T1 and the full effect at T2. Accordingly, to estimate the Group × Time interaction effect, we specified the expected differences in ABI change between the two groups over time as 0, 0.04, and 0.08 at T0, T1, and T2, respectively. The SD of ABI measurements was specified as 0.17 based on longitudinal ABI variability reported in a randomized clinical trial comparing supervised exercise with endovascular revascularization plus supervised exercise in patients with PAD, where ABI SDs ranged from 0.16 to 0.18 (27). A baseline-follow-up correlation coefficient of 0.50 was assumed for repeated ABI measurements in the longitudinal model. The estimated sample size was 116 participants. After accounting for an anticipated attrition rate of 20%, the required recruitment target was 145 participants. The final target sample size was rounded up to 146 participants, with 73 participants allocated to each group. Sensitivity analyses were performed to assess the robustness of the sample size estimation under alternative assumptions for the between-group difference in ABI change (0.05, 0.08, 0.10, and 0.15), with the results presented in Supplementary Table 2.

### 2.12 Statistical analysis

A detailed statistical analysis plan will be finalized before database lock and prior to the initiation of statistical analyses. Trial reporting will follow CONSORT guidelines, with transparent reporting of participant flow, intervention discontinuation, adherence, follow-up status, and outcome completeness (28, 29). Statistical analyses will be conducted according to the intention-to-treat (ITT) principle, including all randomized participants regardless of adherence to or completion of the assigned intervention. All statistical analyses will be performed using R software (version 4.5.2).

#### 2.12.1 Baseline characteristics

Baseline characteristics will be summarized by treatment group. Categorical data will be summarized by numbers and percentages. Continuous data will be summarized as mean (standard deviation [SD]) or median (interquartile range [IQR]), according to data distribution. No statistical hypothesis testing will be performed for comparisons of baseline characteristics.

#### 2.12.2 Outcome analyses

##### 2.12.2.1 Primary estimand and handling of intercurrent events

The primary estimand of this trial is the effect of assignment to an SBDJ-based rehabilitation programme (SBDJ plus ankle pump exercise) compared with ankle pump exercise alone on longitudinal changes in index-limb ABI over the 12-week rehabilitation period among patients with CLTI after lower-limb EVT. This estimand follows the randomized treatment assignment and incorporates prespecified strategies for handling intercurrent events that may influence interpretation of the primary outcome. Discontinuation of the assigned exercise intervention will be addressed using a treatment-policy strategy, and participants will remain analyzed according to their randomized treatment allocation regardless of adherence or discontinuation. Repeat index-limb revascularisation or major/minor amputation will be addressed using a hypothetical strategy because these events represent substantial changes in limb status that may affect the interpretation of the primary outcome. ABI measurements obtained after these events will not contribute to the primary outcome analysis. Death will be considered an intercurrent event rather than ordinary missing data because subsequent outcome assessment is no longer possible and will be summarized separately by treatment group.

##### 2.12.2.2 Analysis of the primary outcome

The primary outcome will be assessed using index-limb ABI measurements obtained at baseline, week 4, and week 12. ABI values >1.40, indicating non-compressible arteries, will be considered uninterpretable and excluded from the primary outcome analysis. The treatment effect for the primary outcome will be estimated using a longitudinal linear mixed-effects model. ABI measurements at the three assessment time points will be modelled as repeated measures. The model will include fixed effects for treatment group, time, group-by-time interaction, study centre, and prespecified clinically relevant covariates, including diabetes status, Rutherford classification, and wound status. A participant-level random intercept will be included to account for within-participant correlation arising from repeated measurements. The primary hypothesis test will be based on the group-by-time interaction, which evaluates whether changes in ABI over time differ between treatment groups over the 12-week intervention period. Statistical inference for fixed effects will use Satterthwaite-adjusted degrees of freedom. Estimated marginal means, between-group differences, 95% confidence intervals, and two-sided *p* values will be reported.

##### 2.12.2.3 Analysis of secondary outcomes

Secondary continuous outcomes (e.g., WHOQOL-BREF, WIQ, SAS, and NRS) will be analysed using linear mixed-effects models for repeated measures. These analyses will be considered exploratory, with no formal adjustment for multiplicity. Effect estimates with 95% confidence intervals will be reported and interpreted in consideration of both statistical significance and clinical relevance.

#### 2.12.3 Intervention adherence analysis

Adherence to the assigned rehabilitation programme will be summarized descriptively by treatment group. The proportion of completed sessions relative to the prescribed sessions will be calculated for each assigned exercise component andsummarized using mean (SD) or median (IQR), as appropriate according to data distribution. The proportion of participants achieving the predefined adherence threshold (≥80% adherence) will be reported as numbers and percentages.

#### 2.12.4 Methods for additional analyses

Prespecified exploratory subgroup analyses will be conducted for the primary outcome (ABI) according to diabetes status, Rutherford classification, wound status, and target lesion location. Treatment effect modification will be assessed by incorporating treatment-by-subgroup interaction terms into the primary longitudinal mixed-effects model. Corresponding effect estimates and 95% confidence intervals will be presented in forest plots. Because the trial is not powered for subgroup comparisons, these analyses will be considered hypothesis-generating and interpreted cautiously.

#### 2.12.5 Missing data and sensitivity analyses

Missing outcome data will be handled using longitudinal linear mixed-effects models based on all available observations under the assumption that data are missing at random. Sensitivity analyses using multiple imputation will be considered to assess the robustness of the findings to missing data. The per-protocol sensitivity analysis will include participants with ≥80% adherence to the assigned rehabilitation programme. Results will be compared with the primary intention-to-treat analysis to assess the robustness of the estimated treatment effects.

## 3 Discussion

For patients with CLTI undergoing lower-extremity revascularization, restoration and maintenance of adequate limb perfusion are essential for symptom relief, reducing repeat interventions, and improving long-term limb outcomes (30–32). Current post-revascularization management primarily relies on pharmacological therapy, risk-factor modification, and vascular surveillance to maintain vessel patency and reduce recurrent events (33). However, long-term vessel patency after EVT remains suboptimal, with reported 1-year primary patency rates of approximately 50%–60% in patients with CLTI (34). Therefore, early rehabilitation strategies that may facilitate recovery after EVT warrant further investigation.

Clinical studies in patients with PAD have demonstrated that exercise training increases brachial artery flow-mediated dilation (FMD), improves calf muscle blood flow, and enhances oxygen extraction capacity (35–37). However, available evidence has predominantly been derived from patients with intermittent claudication, and whether exercise-based rehabilitation influences target-limb haemodynamic status after EVT in patients with CLTI remains uncertain (38–40).

SBDJ is a low-intensity mind–body exercise intervention integrating coordinated movements, breathing regulation, and attentional control, and may represent a suitable rehabilitation exercise for patients with limited exercise tolerance after EVT. Although the mechanisms underlying the effects of SBDJ remain to be elucidated, its comprehensive exercise may influence vascular function and physiological adaptation through autonomic modulation (41, 42). Experimental and clinical studies suggest that low-to-moderate intensity exercise may induce adaptive vascular responses, including alterations in endothelial function, vascular responsiveness, and tissue perfusion capacity (43–45). Furthermore, exercise-related haemodynamic stimuli, including alterations in blood flow and shear stress, may extend these adaptations beyond the directly activated muscle groups to remote vascular beds (43, 46). Studies of upper-extremity exercise have shown that, despite the exercise load being mainly applied to the upper limbs, remote lower-limb vascular beds may exhibit improved endothelial-dependent vasodilation and microvascular function (47). Collectively, these findings provide a physiological rationale for evaluating SBDJ as a rehabilitation strategy in patients with CLTI following EVT.

From a study design perspective, patients after EVT generally receive basic postoperative limb exercises as part of postoperative rehabilitation. Therefore, ankle pump exercise was selected as the common exercise component in both groups of this trial. Ankle pump exercise is a commonly prescribed and feasible postoperative exercise intervention after lower-extremity procedures and may enhance calf muscle pump activity and facilitate lower-limb venous return through repetitive ankle flexion and extension movements (48, 49). In this trial, SBDJ was added to the ankle pump exercise programme to evaluate the potential additional effects of SBDJ-based rehabilitation beyond ankle pump exercise alone.

Regarding outcome selection, ABI was selected as the primary outcome because restoration and maintenance of adequate limb perfusion remain central therapeutic goals after EVT. As a simple, non-invasive, and reproducible measure of arterial haemodynamic status, ABI enables objective assessment of whether SBDJ-based rehabilitation may influence the haemodynamic status after revascularisation. Large-scale CLTI revascularisation studies, including BEST-CLI, have incorporated changes in ABI as an objective marker of post-revascularisation haemodynamic response and reported associations with clinically relevant limb outcomes, including wound healing, amputation-free survival, major adverse limb events, and target lesion revascularisation (50, 51). Previous studies have also reported ABI improvements following exercise training in patients with PAD after lower-extremity revascularisation (52–54). In addition, walking ability, pain, quality of life, and psychological symptoms were included as secondary outcomes to capture broader clinical effects of SBDJ-based rehabilitation programme.

An additional strength of this trial is the integration of a mHealth platform to support the implementation of the home-based rehabilitation programme. Available to both study groups, this platform facilitates standardized exercise delivery, self-monitoring, and follow-up management throughout the intervention period. By supporting sustained engagement with the prescribed rehabilitation programme, this approach may enhance the feasibility and adherence of postoperative rehabilitation, particularly among patients with mobility limitations or barriers to regular healthcare access.

This study has several limitations. This study has several limitations. First, ABI was selected as the primary outcome to assess target-limb haemodynamic changes during the early rehabilitation period after EVT. However, ABI primarily reflects macrovascular haemodynamic status and may not fully capture exercise-induced microvascular adaptations or functional changes associated with rehabilitation. Furthermore, changes in vessel patency after revascularization and arterial incompressibility due to medial calcification may affect the interpretation of ABI measurements. Given the multicentre design of this trial, ABI measurements could be standardized across participating centres, whereas some microvascular assessment methods were limited by equipment availability. Future studies incorporating additional measures, such as transcutaneous oxygen pressure (TcPO_2_), toe-brachial index (TBI), or skin perfusion pressure (SPP), may provide a more comprehensive evaluation of limb perfusion status and vascular responses after rehabilitation. Second, the intervention group received additional SBDJ training and related guidance, which may have resulted in differences in intervention exposure and interaction time between groups. Therefore, the observed effects may reflect the overall SBDJ-based rehabilitation programme. Third, due to the nature of the intervention, participants and intervention providers could not be blinded. Although outcome assessors and statisticians will remain blinded and standardized intervention procedures will be implemented to minimize potential bias, self-reported outcomes may still be influenced by participants' awareness of group allocation. Finally, the relatively short intervention and follow-up period may limit the evaluation of long-term effects of SBDJ-based rehabilitation.
