# Supplementary material for: Sitting Baduanjin-based rehabilitation programme following endovascular therapy in patients with chronic limb-threatening ischemia: protocol for a WeChat mini program-assisted multicentre randomized controlled trial
Source: Front Cardiovasc Med. 2026 Oct 9;13:1921616. doi: 10.3389/fcvm.2026.1921616 (PMC13690648; doi:10.3389/fcvm.2026.1921616)
Supplement: Supplementary file 1 [file Table1.docx]

Supplementary Table 1.The movements of sitting Baduanjin

| The movements of sitting Baduanjin | |
| --- | --- |
| Movement | Main points of the movement |
| Preparation posture | The patient sits on a chair with their legs naturally open or on the bed. |
|  |  |
|  | With the body straight and facing the front, the patient feels calm, at ease, and levitated at the Baihui acupoint. The tongue is pressed against the upper jaw. |
|  |  |
|  | The patient puts their palms in front of the abdomen, closes their eyes, and breathes slightly and peacefully. |
|  |  |
| First movement: “hands in the sky to support Sanjiao” | When inhaling, the patient lifts their hands from their chest to the top of their head. |
|  |  |
|  | When exhaling, the patient drops their hands from both sides of their body to their waist and holds them in front of their abdomen. |
|  |  |
| Second movement: “drawing the bow to shoot the hawk” | The patient reaches their arms out to the sides with their palms down as if they were holding a bow and arrow. They inhale and then exhale as they draw their arms back, bending their elbows and bringing their hands toward their chest. |
|  |  |
|  | They hold for a moment and then release by straightening their arms and returning to the starting position. |
|  |  |
| Third movement: “lifting the arms to regulate the spleen and stomach” | The patient raises the left palm and rotates it through the chest, then brings it upward in front of their face, followed by an inward rotation of the arms to lift it to the upper left side of the head. Simultaneously, they rotate the right palm inward and press it down beside the right hip with their fingertips facing forward. |
|  |  |
|  | The patient then drops their left hand, lifts their right hand simultaneously, and holds their 2 hands in front of their abdomen. |
|  |  |
|  | They repeat the same movement on the opposite side. |
|  |  |
| Fourth movement: “looking backward to relieve the fatigues and 7 injuries” | The patient rotates both arms outward with the palms facing outward, turns their head to the left and back, pauses for a moment, and looks diagonally behind to the left. |
|  |  |
|  | They rotate both arms inward, press them against their hips, move their fingers forward, and look straight ahead. |
|  |  |
|  | They repeat the same movement on the opposite side. |
|  |  |
| Fifth movement: “clearing the heart fire by shaking the head and wagging the tail” | The patient lifts both palms in front of the abdomen and chest, extending them above their head with their fingers facing each other. |
|  |  |
|  | They lower their arms to both sides, placing their palms on top of their thighs. |
|  |  |
|  | They raise the center of gravity slightly, then shift it to the right, tilt their upper body to the right, bend down, and visually observe the surface of their right foot. |
|  |  |
|  | They shift the center of gravity to the left while rotating their upper body from right to front and left, visually observing their right heel. |
|  |  |
|  | They shift the center of gravity to the right, shake their head backward, slightly retract their jaw, and look ahead. |
|  |  |
|  | The right-side action is the same as the left-side action but in the opposite direction. |
|  |  |
| Sixth movement: “move 2 hands down to the leg to strengthen the kidney and lumbar area” | The patient raises both arms forward and upward, with their palms facing forward. |
|  |  |
|  | They press their palms against their chest with the elbows bent, palms facing downward, and fingers facing each other. |
|  |  |
|  | They rotate both arms outward with their palms facing upward and then insert the palm fingers under the armpits. |
|  |  |
|  | They rub their palms inward along the spine to the kidney area and then rub them back and forth. |
|  |  |
|  | They return their hands to the front of the abdomen through the body. |
|  |  |
| Seventh movement: “tighten fists to gather strength” | The patient holds both fists at the waist, with the thumb inside and the fist facing upward, and looks straight ahead. |
|  |  |
|  | They inhale and hold their fists, exhale and clench their left fist with a furious gaze, keep their eyes wide open, grip firmly, inhale, and retract. |
|  |  |
|  | They exhale and clench their right fist with a furious gaze, keep their eyes wide open, grip firmly, inhale, and retract. |
|  |  |
| Eighth movement: “rotate shoulders to enhance yang and qi” | The patient lowers both arms naturally, contracts the abdomen, and expands the chest. |
|  |  |
|  | They inhale to lift their shoulders and rotate them inward and exhale to lower their shoulders. |
|  |  |
|  | They inhale to lift their shoulders and rotate them outward and exhale to lower their shoulders. |
|  |  |
| Closing posture | The patient wipes their face, combs their hair, rubs their ears, and taps their left and right elbow creases. |

Supplementary Table 2. Sensitivity analysis of required sample sizes across different assumed between-group differences in ABI change

| Assumed between-group ABI difference at 12 weeks | Required sample size (total) | Interpretation |
| --- | --- | --- |
| 0.05 | 288 | Small effect scenario |
| 0.08 | 116 | Primary assumption |
| 0.1 | 76 | Moderate effect scenario |
| 0.15 | 36 | Optimistic upper-bound scenario |
